# Characteristics and mortality determinants of COVID-19 patients undergoing hemodialysis

**DOI:** 10.3906/sag-2006-54

**Published:** 2021-04-30

**Authors:** Savaş SİPAHİ, Hamad DHEİR, Aysel TOÇOĞLU, Melike BEKTAŞ, Seyyid Bilal AÇIKGÖZ, Ahmet Cihat GENÇ, Fuldem MUTLU, Mehmet KÖROĞLU, Ali Fuat ERDEM, Oğuz KARABAY

**Affiliations:** 1 Department of Internal Medicine, Division of Nephrology, Faculty of Medicine, Sakarya University, Sakarya Turkey; 2 Department of Internal Medicine, Faculty of Medicine, Sakarya University, Sakarya Turkey; 3 Department of Internal Medicine, Faculty of Medicine, 19 Mayıs University, Samsun Turkey; 4 Department of Radiology, Faculty of Medicine, Sakarya University, Sakarya Turkey; 5 Department of Microbiology, Faculty of Medicine, Sakarya University, Sakarya Turkey; 6 Department of Anaesthesiology, Faculty of Medicine, Sakarya University, Sakarya Turkey; 7 Department of Infectious Diseases and Clinical Microbiology, Faculty of Medicine, Sakarya University, Sakarya Turkey

**Keywords:** Chronic renal failure, hemodialysis, COVID-19

## Abstract

**Background/aim:**

The COVID-19 infection, which started in Wuhan City, China, in December 2019, turned into a pandemic in a very short time, affecting mainly the elderly and those with serious chronic illnesses. COVID-19 infections have been observed to have a high mortality rate, especially in patients undergoing maintenance hemodialysis.

**Materials and methods:**

Forty-two patients over 18 years of age who underwent a maintenance hemodialysis program at our unit, who tested positive for COVID-19 by PCR from nasopharyngeal swabs, and/or who were observed to have disease-related signs in their CTs were included in the study.

**Results:**

In this study, 23 of 42 patients receiving hemodialysis support in our clinic were included. The median age was 67 years old (min: 35; max: 91 years), and all of our patients had primary hypertension and other comorbidities. Their clinical evaluation showed that dry cough (47.8%) and shortness of breath (47.8%) were the most common symptoms. Fever was less pronounced (30.4%). The median time from the onset of symptoms to hospitalization was 1 day (min: 0; max:), and the time from hospitalization to death was 18 days (min: 1; max: 22). Transfer from the inpatient ward to the ICU took a median of 7 days (min: 1; max: 13). Among the 23 patients, 3 died during follow-up, and 20 were discharged with full recovery. Baseline ferritin, procalcitonin levels, and CRP/albumin rates were higher, and neutrophil/lymphocyte levels were lower in patients who eventually died. In these patients, despite being nonsignificant, there were more diabetic patients, and the D-dimer levels were higher than 1000 ugFEU/L.

**Conclusion:**

The COVID-19 infection is associated with increased mortality in chronic kidney diseases patients. Despite being nonsignificant, there was a trend towards increased mortality in patient with diabetes, D-dimer levels >1000 ugFEU/L, higher ferritin and prokalsitonin levels, an increased CRP/albumin ratio, and a lower neutrophil/lymphocyte ratio.

## 1. Introduction

The COVID-19 infection, which started in Wuhan City, China in December 2019 and which has become a pandemic in a very short time, affects the elderly and those with serious chronic illnesses [1]. The COVID-19 pandemic has created an increased burden and stress on healthcare systems because it has no known treatment or vaccine yet [2]. The infection risk is greater in individuals with chronic renal failure (CRF) and especially in patients receiving dialysis treatment. Infections are the second most common cause of death, following cardiovascular diseases [3].

Treatment of dialysis patients diagnosed with COVID-19 infection leads to some additional difficulties, especially for patients undergoing hemodialysis in anin-center unit [4]. These patients are vulnerable against infections due to their immunocompromised status and, thus, clinical symptoms may vary, as well, leading the symptoms to be misleading in some patients. Medical staff in hemodialysis centers may also significantly increase the risk of infecting patients and family members [5]. 

The COVID-19 infection progresses with a high mortality rate in patients undergoing hemodialysis, in peritoneal dialysis patients, and in patients developing acute renal failure due to COVID-19 infection [4]. It would therefore be interesting to observe whether the clinical course of patients with COVID-19 undergoing a dialysis program is different from that of other COVID-19 patients.

It is crucial for units with maintenance hemodialysis (MHD) patients to rapidly develop protective and preventive strategies during pandemics. Thus, as part of these strategies, predictive indicators for mortality may contribute to patient survival and the shortening of the hospitalization period. 

## 2. Materials and methods

### 2.1. Overall COVID-19 strategy

At the time of this study, 852 patients were being treated in the chronic hemodialysis units in all public and private dialysis centers in the city of Sakarya (overall population: 1.03 million people). Since the onset of the disease and since the report of the first case in Turkey, (11.03.2020), the Sakarya University Medical Faculty Department of Nephrology contacted all dialysis centers around the city. Before patients were taken via transportation services across the city, we used a digital thermometer to measure their body temperature before they entered vehicles. Their symptoms were also examined, and patients whose temperature was over 38 °C and/or who were identified as having symptoms of COVID-19 were not allowed into the vehicles. Instead, they were taken to our hospital via ambulance. The same procedures were conducted in the entrances and halls of all hemodialysis centers, as well. 

Separate rooms within the dialysis center was reserved for COVID-19 patients to undergo hemodialysis. We then distributed all the noninfected patients in the dialysis center to other centers to prevent possible crosscontamination with patients infected with COVID-19. This was a strategy used in order to gather all of the patients who were symptomatic and/or were diagnosed only at our center (single center) to protect other surrounding centers. 

In order to diagnose COVID-19, we received nasopharyngeal swab (NPSW) from all of the MHD patients who had disease-related clinical findings and who were referred to the emergency service of our hospital or who were admitted to the COVID outpatient clinic for PCR analysis and chest computed tomography (CT) (lower dose of 80%).

All of the MHD patients who were thought to have COVID-19 diagnosis were hospitalized. A treatment containing oseltamivir, hydroxychloroquine, and azithromycin–if necessary– was applied for 5 days. Daily low-molecular-weight heparin (LMWH) was administered during the intradialytic period. When the patients had a negative result for NPSW-PCR during hospitalization, the test was repeated 24 h later. NPSW-PCR tests were also repeated on the 14th day for those who had a positive result. The patients who tested negative twice for NPSW continued treatment and follow-up if their CT findings were compatible with COVID-19. 

### 2.2. Follow-up

Their oxygen needs and oxygen saturations were followed. The patients whose oxygen saturation was below 80% and who had tachypnoea were followed up in the intensive care unit. Patients who had a clinical recovery were discharged after receiving hemodialysis regularly for 14 days in our outpatient hemodialysis center. Hemodialysis treatment of the patients who had negative result for NPSW-PCR 14 days later was allowed to continue in our centers. 

Li et al. mentioned referring patients to a hospital designed for COVID-19 [5]. However, no outcomes from another center following hemodialysis patients with such an application have yet been reported as of the writing of this article.

### 2.3. COVID positive patient diagnosis and treatment strategy

At the time of the analysis of patient data, renal replacement treatment was applied to 98 patients to COVID-19 diagnosed or suspected cases (23.04.2020). Patients over 18 years of age who had CRF and thus had been receiving hemodialysis 3 days and for 4 h a week for at least 3 months were included in the analysis. Among these patients, those who tested positive for COVID-19 as a result of NPSW, which was received for the PCR analysis and/or had disease-related signs in their CT, were included in the study. Patients who had a history of hepatitis, active infectious/inflammatory disease or acute ischemic vascular disease within the last 3 months, or chronic liver disease and malignancy were excluded from the study. Twenty-three patients were included in the analysis. 

Demographic (sex, date of birth, onset of dialysis, cause of CRF, presence of comorbidities, height, weight, hepatitis status, and medications) and laboratory data (hemogram, CRP, procalcitonin, ferritin, glucose, urea, creatinine, Na, K, P, Ca, Mg uric acid, total protein, albumin, ALT, AST, ALP, D-dimer, LDH, blood gas analyses, troponin, pt, aptt, COVID-19 PCR, and Elisa results), as well as intensive care unit data, adjunctive therapies, and ventilator support type/features), were also included in the analysis. 

### 2.4. Laboratory tests

Complete blood count, CRP, procalcitonin, ferritin, glycemia, urea, creatinine, Na, K, Ca, P, Mg, uric acid, total protein, albumin, ALT, AST, ALP, activated partial thromboplastin time, prothrombin time, D-dimer, lactate dehydrogenase, troponin levels, and blood gas analysis were measured with a central laboratory biochemistry analyzer (ICT [ISE] Module of ARCHITECT c16000, Abbott Laboratories, Abbott Park, IL, USA).

### 2.5. RT-PCR COVID test

Combined nasopharynx and oropharynx swab samples were collected by Dacron swab and immediately placed in viral transport medium. They were then delivered to our central laboratory and kept at a temperature of between 2–8 °C. After processing the samples in the microbiology laboratory, the samples were stored at a negative pressure chamber with 3rd level biosafety. A Bio-Speedy Viral Nucleic Acid Isolation Kit (Bioeksen, İstanbul, Turkey) was used for total nucleic acid isolation from the specimens. The isolation procedure was carried out according to the recommendations of the manufacturer. A Bio-Speedy COVID-19 RT-qPCR Detection Kit (Bioeksen) was used for the RT-PCR assays. The PCR amplification and evaluation of the results were carried out according to the manufacturer’s recommendations.

### 2.6. CT measurements

The same expert retrospectively evaluated unenhanced chest CT scan examinations, which were performed for scanning at 5-mm section thickness and at 100-120 KV intervals and low dose option (80%) by adjusting the automatic exposure mA values appropriately in 64-slide CT devices (Toshiba Aquillion 64, Japan). 

### 2.7. Statistical analysis

All data were assessed by using SPSS version 21.0 (SPSS, Chicago, IL, USA). All numerical variables were reported as median (minimum value–maximum value), and categorical parameters were reported as number and percent. Patients were compared with nonparametric analysis. A Mann–Whitney U test was used for numerical variables, whereas a chi-square test was used to assess categorical variables. A P value less than 0.05 was accepted as statistically significant.

## 3. Results

This descriptive crosssectional study was conducted among 23 COVID-19 infected MHD patients between March 14 and April 22 of 2020. In total, 98 patients receiving renal replacement treatment in our hospital due to COVID-19 prediagnosis were evaluated. Among these patients, 42 were included in the chronic haemodialysis program. Ten patients were excluded due to catheter infection, urinary system infection, cholecystitis and hypervolemia diagnoses; 6 patients were excluded due to nontypical pneumonia signs for COVID-19; 3 patients due to malignancy were excluded from the study. Twenty-three patients included in the hemodialysis program were diagnosed with COVID-19 and immediately started treatment and follow-up. The dialysis duration of the patients was 45.0 (3.5–158.8) months.

Table 1 shows the demographic characteristics of the patients. Their median age was 67 (35–91) years, and 23 (100%) of them had comorbidities, hypertension being the most common.

**Table 1:** Demographic characteristics of all patients.

	n	%
Age, years, median (range)	67 (35-91)	100
Male	14	60.9
Female	9	39.1
History of exposure to COVID patient	11	47.8
Comorbidity		
Hypertension	23	100
Cardiovascular disease	12	52.2
Chronic obstructive pulmonary disease	3	13.0
Diabetes	11	47.8
Other chronic diseases	1	4.3

During follow-up, dry cough (47.8%) and shortness of breath (47.8%) were the most common symptoms (Table 2). The median arterial oxygen saturation was 95 (range: 89–98%). Fever was observed, but the percentage was less than expected (30.4%). The median time from the onset of symptoms to hospitalization was 1 day (min: 0; max: ), and the time from hospitalization to death was 18 days (min: 1; max: 22). Transfer from the inpatient ward to the ICU took a average of 7 days (min: 1; max: 13). The median time elapsed from the onset of symptoms to death was 19 days (min: 2; max: 23). 

**Table 2:** Clinical manifestations and physical signs of all patients.

Items	n	%
Fever >39°C	7	30.4
Cough	11	47.8
Dyspnoea	11	47.8
Myalgia or fatigue	3	13.0
Headache	3	13.0
Diarrhoea	1	4.3

Lymphopenia was observed in 10 patients (43.5%). NPSW-PCR was positive at least once, and radiological signs were identified in 5 out of 23 patients. Nineteen patients had typical radiological signs (ground-glass appearance, fine reticular opacities, vascular thickening) (Table 3). 

**Table 3:** Laboratory tests of all patients.

Items	Median (min-max)
White blood cell count × 109/L	8510 (3400-26600)
Lymphocyte count × 109/L	1120 (220-7880)
Platelets × 109/L	162000 (30710-448000)
C-reactive protein, mg/L	52.9 (1.7-162.9)
Procalcitonin, ng/ml	0.51 (0.02-100.0)
D-Dimer, ugFEU/L	1210(138-11000)
Albumin, gr/L	31.6(20.4-37.2)
Neutrophil/Lymphocyte ratio	3.7(1.1-36.0)
CRP/albumin ratio	1.75(0.06-5.06)

A treatment containing oseltamivir, hydroxychloroquine, and azithromycin–if necessary– was prescribed to all the patients diagnosed with COVID-19 for 5 days. On the intradialytic days, LMWH was administered. Patients who were nonresponsive to this treatment approach continued the treatment for additional 10 days, and favipiravir was added to the treatment protocol (Table 4). Prolongation of QTc was not identified in any of the patients taking hydroxychloroquine and azithromycin. 

**Table 4:** Treatment regimen of all patients.

Treatment	n	%
Oseltamivir	21	91.3
Hydroxychloroquine	23	100
Azithromycin	19	82.6
Favipiravir	5	21.7
Methylprednisolone	2	8.7

Among the 23 patients, 3 died during the follow-up, and 20 were discharged with full recovery. Both patients who died and survivors were covered in terms of their demographics, laboratory tests, and intensive care onset times (Table 5). Despite being nonsignificant, baseline ferritin, procalcitonin levels, and CRP/albumin rates were higher and neutrophil/lymphocyte levels were lower in patients who died. Among these patients, there were more diabetic patients, and D-dimer levels were higher than 1000 ugFEU/L.

**Table 5:** Possible factors affecting mortality in MHD patients with COVID-19 infection.

Items	Mortality groupn = 3	Survival groupn = 20	P
Diabetes mellitus (n)	3 (27.3%)	8 (72.7%)	.093a
Admission to intensive care unit (day)	18 (1-22)	6 (2-15)	.400b
Ferritin, µg/L	2000 (652-3590)	583 (57-3004)	.094b
D-dimer (>1000) (n)	3 (23.1%)	10 (76.9%)	.240 a
D-dimer,ugFEU/L	1530 (1150-11500)	1200 (138-4760)	.265 b
Lactate dehydrogenese (LDH)	222(169-366)	280(141-421)	.586 b
Lymphocyte count, × 109/L	1070 (940-1370)	1165 (220-7880)	.966 b
Neutrophil/ Lymphocyte ratio	2.77 (2.72-3.49)	4.50 (1.07-36.0)	.160 b
Serum albumin	31.0(28.4-33.8)	31.8(20.4-37.2)	1,00 b
CRP/albumin ratio	2.4 (1.7-3.2)	1.5 (0.1-5.1)	.509 b
Procalcitonin	1.3(1.0-100)	0.4(0-5.0)	.082 b
Uric acid, mg/dL	5.4 (4.7-6.0)	4.4 (2.7-7.0)	.513 b

a: chi-square test, b: Mann–Whitney U test.

## 4. Discussion

The coronavirus (CoV) family is a large virus family that is commonly observed in society. It exhibits cold-like symptoms and may cause conditions ranging from self-limited mild infection to more serious cases such as Middle East Respiratory Syndrome (MERS) and Severe Acute Respiratory Syndrome (SARS) [6].

CoVs have a variety of subtypes that infect humans, and they can be transmitted from easily person to person. However, some types are known to be carried in animals and can possibly be transmitted from animals to humans, thus resulting in severe diseases [6].

On December 31st, 2019, the China World Health Organization Country Office reported pneumonia cases with unknown etiology in the city of Wuhan in Hubei province. The patients had fever, dyspnoea, and radiologic signs that were compatible with bilateral pulmonary pneumonic infiltration [7].

On January 7th, 2020, the agent was identified as a new Coronavirus (2019-nCoV), one that had not been identified in humans before. The name 2019-nCoV was then accepted as COVID-19, and the virus was named as SARS-CoV-2 due to its similarity with SARS CoV. The disease has spread rapidly due to its characteristic transmission from person-to-person [8].

MHD patients have a higher risk of getting COVID-19 and suffer from its complications. The majority of these patients are elderly and have multiple comorbidities, including but not limited to cardiovascular diseases, diabetes mellitus, hypertension, and chronic pulmonary diseases. In addition, they have a weaker immune systems and a lower resistance against infections [3]. MHD patients have 3 weekly visit to dialysis units and share the same environment during hemodialysis, so this increases the risk of the disease transmission [4].

In both the CDC and centers where the pandemic is intense, it is recommended that patients with symptoms undergo hemodialysis in specific rooms and have separate sessions. The number of sessions should also be reduced [4,5].

Thus, the same rule was applied in our center with a dedicated room for COVID-19 positive patients. Later, we referred all of the patients in the dialysis center to other centers and prevented their possible encounter with patients infected with COVID-19. We also gathered all patients who were symptomatic and/or were diagnosed in our center (single center) and thus protected patients from other centers. We believe that by doing this, we prevented more patients in our city from becoming infected. 

The mean age of the patients was 66.7 ± 12.0 years, and all of them had comorbidities– hypertension and cardiovascular diseases being the most prevalent (100%). In the literature, the most common symptoms during admission are fever, fatigue, and dry cough. Other less common symptoms are myalgia, chest tightness, shortness of breath, nausea, vomiting, and diarrhoea [9]. In our patient population, the most common symptoms were dry cough and shortness of breath. Fever was less observed, which might be due to the inadequate fever response of the hemodialysis patients to infection. This is due to the fact that MHD patients have compromised immune systems because of the uremic state, which is often accompanied by significant comorbidities. The fact that shortness of breath and cough were the main symptoms is important in terms of hypervolemia, which is frequently encountered among patients undergoing hemodialysis in the differential diagnosis. 

The NPSW-PCR test for viral nucleic acid is accepted as the gold standard method to identify SARS-CoV-2. However, it has also been reported that it leads to false negative results due to technique and because of the difficulty of application and standardization of the kits [9]. This was the initial reason why we performed CT scans on all of the patients to confirm the findings. Multiple bilateral ground-glass opacities/consolidations were considered as a sign of typical, viral pneumonia findings for COVID-19 [9,10]. 

NPSW-PCR was positive in 7 out of 23 patients. In addition, 19 patients had typical radiological signs (ground-glass appearance, fine reticular opacities, vascular thickening). Ai T et al. stated that, although RT-PCR plays a crucial role for hospitalization and isolation, it is ineffectual in controlling the disease due to its low sensitivity and time requirement. The authors indicated that when the sensitivity of a thoracic CT is evaluated, especially with serial CT images in diagnosis of COVID-19, it is quite high and provides an opportunity for identifying 93% of patients with NPSW-PCR negative results much earlier[11]. We believe that a combination of contact history, clinical findings, and imaging findings in patients with negative NPSW-PCR test results is more beneficial in diagnosing COVID-19 [9,10,12]. (43.5% and 59.1%, respectively) In another article, Zhou et al. reported advanced age, a higher SOFA score, and a D-dimer above 1 μg/mL as parameters in identifying patients with a potentially poor prognosis during early periods [13]. 

Patients dying due to COVID-19 go through a rapid clinical course. The overall fatality rate of patients with confirmed COVID-19 in the Italian population, based on data up to March 17, was 7.2% (1625 deaths/22512 cases) [14]. In our study, the mortality rate was much higher than the 2.7% reported for the general population in Turkey. In our patients, the median time that elapsed from the onset of symptoms to hospitalization was 1 day, and the median time that elapsed from hospitalization to death was 18 days. It took an average of 7 days for the patients to be transferred to the ICU. Among the 23 patients, 3 died (13%) during follow-up, and 20 were discharged with full recovery. 

This rapid course requires rapid treatment, however, there is no known treatment approach for COVID-19 yet [2]. Almost every country and every clinic applies its own protocols. The National Health Commission of the PRC Guideline recommends inhalation of interferon α and lopinavir/ritonavir as an aerosol. The specific therapeutic value and safety of lopinavir/ritonavir have been investigated in COVID-19 patients (ChiCTR2000029308). It has been reported that remdesivir also leads to successful outcomes in COVID-19 patients. Multicentered clinical trials conducted in China have revealed that chloroquine phosphate is slightly effective on pneumonia related with COVID-19 [15]. Tang et al. recommended treating patients with an anticoagulant, considering the change in D-dimer levels [16]. 

We administered oseltamivir 1 × 75 mg and oral hydroxychloroquine at the end of dialysis at a dose of 200 mg every other day to the patients without pneumonia who were confirmed as having COVID-19 and a oseltamivir 1 × 75 mg oral hydroxychloroquine at the end of dialysis, oral azithromycin 200 mg every other day, an azithromycin 500 mg tablet on the first day and 250 mg per day for the following 4 days to patients with pneumonia. Additionally, we used favipiravir 2 × 1600 mg as loading and 2 × 600 mg as maintenance in patients with severe cases who did not effectively respond to the aforementioned treatment approach. We applied LMWH in all the hemodialysis cases during and outside dialysis, based on D-dimer and BMI (if D-dimer <1000 ugFEU/L and/or BMI <40 kg/m2, enoxaparin 40 mg; if D-dimer >1000 ugFEU/L and/or BMI >40 kg/m2, enoxparin 60 mg). 

MHD patients have a higher risk of COVID-19 and its complications. These patients also have a higher mortality risk than the general population (general population: 2.7%; hemodialysis population: 13%). 

Our study has certain limitations. Since we conducted the present study in a relatively smaller patient group, some of our determinations unfortunately do not go further than prediction, and there is a lack of statistical significance. Despite being nonsignificant, there was a trend towards increased mortality in patient with diabetes, D-dimer levels >1000 ugFEU/L, higher ferritin and prokalsitonin levels, an increased CRP/albumin ratio, and a lower neutrophil/lymphocyte ratio.

## Ethical standard

This study was conducted in accordance with the Declaration of Helsinki. All of the participants signed written informed consent for the study, which was approved by the Ethics Committee of Sakarya University Medical Faculty. The code is 71522473/050.01.04/124 (20.03.2020).
